# A Genome-Wide Analysis of *FRT*-Like Sequences in the Human Genome

**DOI:** 10.1371/journal.pone.0018077

**Published:** 2011-03-23

**Authors:** Jeffry L. Shultz, Eugenia Voziyanova, Jay H. Konieczka, Yuri Voziyanov

**Affiliations:** 1 School of Biological Sciences, Louisiana Tech University, Ruston, Louisiana, United States of America; 2 Harvard University/Broad Institute, Cambridge, Massachusetts, United States of America; Sanford-Burnham Medical Research Institute, United States of America

## Abstract

Efficient and precise genome manipulations can be achieved by the Flp/*FRT* system of site-specific DNA recombination. Applications of this system are limited, however, to cases when target sites for Flp recombinase, *FRT* sites, are pre-introduced into a genome locale of interest. To expand use of the Flp/*FRT* system in genome engineering, variants of Flp recombinase can be evolved to recognize pre-existing genomic sequences that resemble *FRT* and thus can serve as recombination sites. To understand the distribution and sequence properties of genomic *FRT*-like sites, we performed a genome-wide analysis of *FRT*-like sites in the human genome using the experimentally-derived parameters. Out of 642,151 identified *FRT*-like sequences, 581,157 sequences were unique and 12,452 sequences had at least one exact duplicate. Duplicated *FRT*-like sequences are located mostly within LINE1, but also within LTRs of endogenous retroviruses, Alu repeats and other repetitive DNA sequences. The unique *FRT*-like sequences were classified based on the number of matches to *FRT* within the first four proximal bases pairs of the Flp binding elements of *FRT* and the nature of mismatched base pairs in the same region. The data obtained will be useful for the emerging field of genome engineering.

## Introduction

Site-specific tyrosine recombination systems, such as Flp/*FRT*, Cre/loxP and related systems catalyze conservative DNA rearrangements between their cognate recombination target sites [1], [2], [3]. By manipulating the relative location and orientation of the recombination target sites, genome rearrangements catalyzed by recombinases can include integration, excision, inversion or recombinase-mediated cassette exchange (RMCE). As these recombination systems are active in all cell types tested, they became popular molecular tools for directed genome rearrangements, including specific DNA insertions or targeted DNA deletions in chromosomes, DNA translocation, gene replacement as well as expression of proteins from selected chromosomal locales, excision of large chromosomal DNA segments for sequencing, rescue of pathogenic islands and production of biofactories [1], [2], [4], [5], [6].

For a directed recombination event to occur using current technology, native recombination target sites must be pre-introduced into a genome locale of interest by homologous, random or vector-mediated recombination. This requirement limits application of the site-specific tyrosine recombination systems in genome engineering mainly to modeling experiments. To expand the use of tyrosine recombination systems, variants of the recombinases have to be evolved to recognize pre-existing genomic sequences that resemble the respective native target sites. These pre-existing genomic sequences paired with the evolved recombinase variants can be then used in genome engineering experiments. Advances in changing target DNA specificity of Cre recombinase [7], [8], [9], [10], Flp recombinases [11], [12], [13], [14] and recently λ Int [15] create an opportunity to develop genome engineering tools based on tailor-made variants of tyrosine recombinases.

For genome engineering experiments to be successful, one needs data on sequence properties and distribution of target-like sites within the genome of interest. A pilot analysis of *FRT*-like sequences in selected contigs of the human genome and some bacterial and viral genomes using the TargetFinder program [14] indicated that the frequency of *FRT*-like sequences in a genome depends on the genome's G/C content and can range from 1 *FRT*-like sequence per ∼1 Mb in a genome of thermophilic bacteria to 1–2 *FRT*-like sequences per 10 Kb in mammalian genomes [[14] and Y.V., unpublished]. The parameters for identifying *FRT*-like sites incorporated into TargetFinder were based mainly on the results of exhaustive mutational analysis of each position of Flp binding elements of *FRT* and its spacer region [16], [17] and the results of our target-linked Flp evolution experiments (Y.V., unpublished). As shown in the present work, the search parameters incorporated into TargetFinder were not always able to correctly identify functional *FRT*-like sequences – the ones that can serve as recombination targets for the evolved Flp variants. In addition, we noticed that TargetFinder worked efficiently only if the genomic DNA string analyzed was about couple of million base pairs long, making the program difficult to use for analyzing long genomic contigs. In fact, TargetFinder required about one month of machine time to scan the entire human genome for *FRT*-like sequences. Therefore, we fine-tuned the parameters for identifying *FRT*-like sites based on the evolvability of Flp variants specific for genomic *FRT*-like sites, incorporated these parameters into the search engine of a newly developed bioinformatics package TargetSiteAnalyzer and performed genome-wide analysis of *FRT*-like sites in the human genome (NCBI build 36.3). Like TargetFinder, TargetSiteAnalyzer is written in Java. TargetSiteAnalyzer is composed of three programs that sequentially scan all genomic contigs and sort the identified *FRT*-like sequences into groups and subgroups. TargetSiteAnalyzer needs only several hours to perform the analysis of the human genome.

We found that functional *FRT*-like sequences can be found in the human genome roughly every 5 kb, although we identified genomic regions with a significantly lower density of *FRT*-like sequences. The identified *FRT*-like sequences were grouped into a limited number of unique classes and subclasses that have common sequence patterns. TargetSiteAnalyzer can be modified to search for target-like sequences for other site-specific recombinases. Our work will be useful for the emerging field of genome engineering.

## Results

### Sequence features of functional genomic *FRT*-like sites

**Figure 1:**
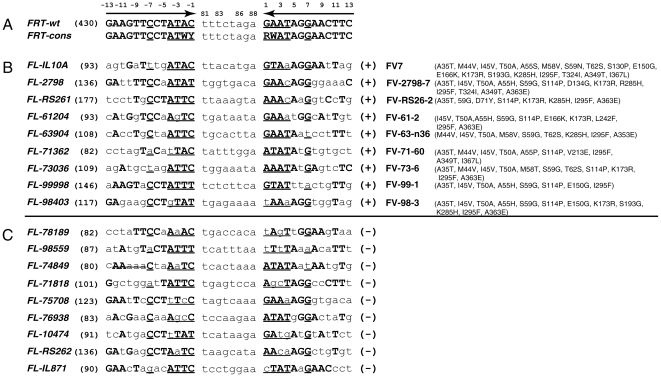
*FRT* (A), successful *FRT*-like sequences (B) and unsuccessful *FRT*-like sequences (C). *FRT*-wt  =  wild-type *FRT*; *FRT*-cons =  consensus *FRT* sequence that reflects nucleotide changes known to be tolerated by Flp variants. *FRT*-cons served as a query to scan the human genomic sequences to identify *FRT*-like sequences. *FRT*-like sequences *FL-IL10A, FL-2798* and *FL-IL871* are from the human interleukin-10 gene; *FL-RS261* and *FL-RS262* are from human ROSA26 locus; *FL-61204*, *FL-63904*, *FL-71362*, *FL-71818*, *FL-73036*, *FL-74849*, *FL-75708*, *FL-76938* and *FL-78189* are from the human beta-globin gene; *FL-98403* and *FL-99998* are from mouse Rosa26 locus; *FL-10474* is from the mouse tyrosinase gene. Numbers in brackets indicate the scores the *FRT*-like sequences have according to the program TargetFinder [14]. Flp variants able to recombine the respective *FRT*-like sequences with at least 10% efficiency are shown next to the successful *FRT*-like sequences. Flp variant FV7 able to recombine *FL-IL10A* was reported earlier [14].

Using the search program TargetFinder [14], we identified 18 model *FRT*-like sequences: in the human interleukin-10 gene, the human beta globin gene, the human and mouse ROSA26 regions and in the mouse tyrosinase gene (Figure 1). According to TargetFinder, the identified *FRT*-like sequences had high scores and were expected to be functional. To evolve variants of Flp recombinase specific for the *FRT*-like sequences shown in Figure 1B, we used a one-step recombinase evolution approach, which includes one round of DNA shuffling between a library of Flp genes mutated at position 59 (S59G) and randomized at positions 55 and 58, and the FV7 gene, which served as a source of core mutations seen in Flp variants with evolved target specificity [14]. The DNA shuffling was followed by selection for Flp variants with desired target specificity using our standard deletion reporters [11], [14]. The choice of amino acids in Flp to be mutated (55, 58 and 59) was based on the Flp/*FRT* co-crystal [18] and on our observations that the first four base pairs of Flp binding elements of *FRT* (positions −1 to −4 and 1 to 4, Figure 1A), which interact with amino acids at positions 55, 58 and 59, are the most critical in Flp/*FRT* recognition (E.V. and Y.V., unpublished).

**Figure 2:**
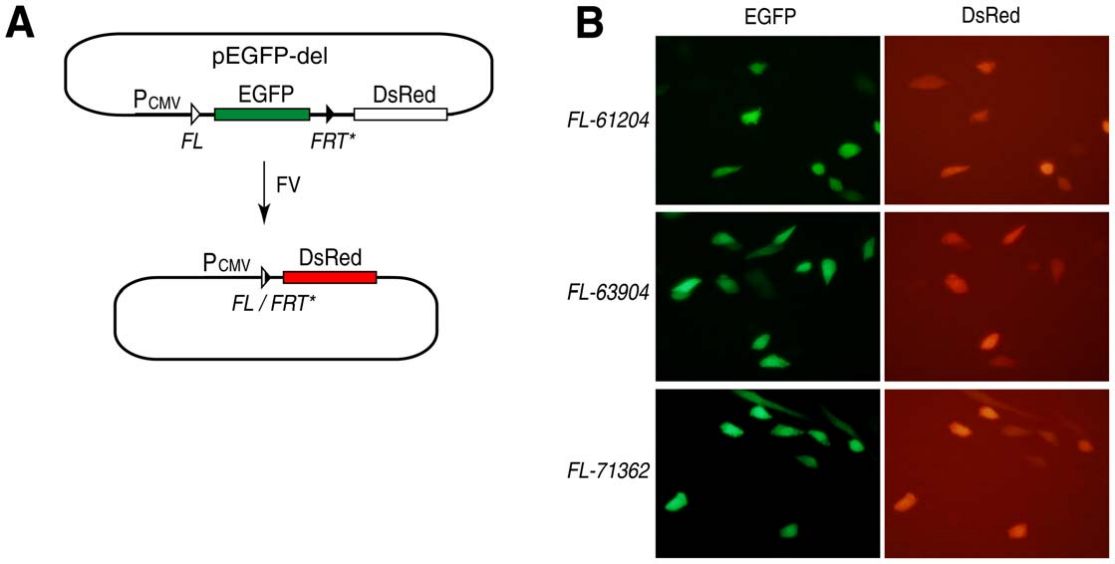
Evolved variants of Flp recombinase are active in mammalian cells. **(A)** Schematics of the deletion assay used to test Flp variants in CHO cells. An episomal reporter pEGFP-del bears the EGFP expressing cassette followed by a promoterless DsRed gene. The EGFP gene is flanked by two recombination sites: genomic *FRT*-like sequence (either *FL-61204*, *FL-63904*, or *FL-71362*; these sites are shown as ‘*FL*’) and modified *FRT* (shown as FRT*), in which its native spacer was substituted with the spacer from the respective *FRT*-like sequence. The choice of genomic *FRT*-like sequences and modified *FRT* as recombination partners for the evolved Flp variants was suggested by the results of our earlier experiments with Flp variant FV7 which showed that this combination of recombination sites works the best in the excision assay [14]. (**B)** Evolved Flp variants efficiently recombine their respective *FRT*-like sequences in CHO cells.

Despite extensive experimenting, we were only able to evolve Flp variants for a half of the selected *FRT*-like sequences (marked ‘+’ in Figure 1B), which we consider true functional genomic *FRT*-like sequences. Flp variants evolved to recombine these genomic *FRT*-like sequences were tested in *E. coli* and showed at least 10% efficiency in a standard deletion assay [11]. To demonstrate that true functional genomic *FRT*-like sequences can also serve as substrates for the respective Flp variants in mammalian cells, we tested Flp variants that can recombine *FRT*-like sequences located in the human b-globin locus (*FL-61204*, *FL-63904*, and *FL-71362*, Figure 1B) in CHO cells (Figure 2). For this, we used an episomal reporter pEGFP-del, which bears the EGFP expressing cassette followed by a promoterless DsRed gene. The EGFP gene was flanked by two recombination sites: either *FL-61204* or *FL-63904* or *FL-71362* and modified *FRT*, in which its native spacer was substituted with the spacer from the respective *FRT*-like sequence to allow recombination between the two sites (Figure 2A). Successful recombination between the recombination sites that flank the EGFP gene leads to the activation of expression of the DsRed gene (Figure 2A). As the results show, all three Flp variants tested are active on their respective genomic *FRT*-like sequences in mammalian cells (Figure 2B).

**Figure 3:**
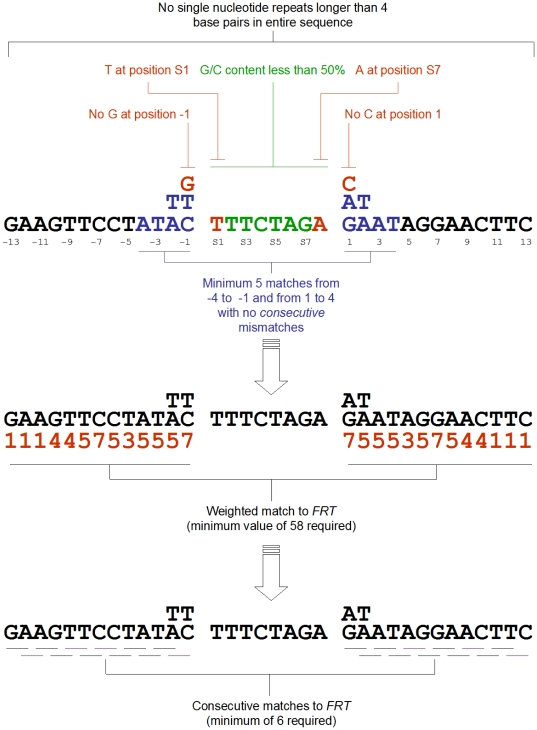
*FRT*-like sequence identification requirements. See text for details.

The comparative analysis of sequence features of functional genomic *FRT*-like sequences (Figure 1B) vs. non-functional ones (marked ‘–’ in Figure 1C) suggested a set of rules that can distinguish between the two groups. The three most important rules that describe functional genomic *FRT*-like sequences are the following: (1) within the proximal 4-bp DNA segments of both binding elements of an *FRT*-like sequence (‘proximal–8 region’; positions −4 through −1 and 1 through 4, which make eight base pairs in total, Figure 1A), there should be at least five matches with the corresponding base pairs of *FRT*; (2) there should not be consecutive mismatches within the same 4-bp DNA segments; (3) at least one binding element should have a match at position 7. In addition, we do not consider genomic *FRT*-like sequences as functional ones if they have mismatches at positions −1 and 1 simultaneously or a ‘G’ at position −1 or a ‘C’ at position 1. We also noted that a functional genomic *FRT*-like sequence should have at least 5 matches in one of its binding elements and at least 6 consecutive matches within both of its binding elements. The above rules along with a scoring system (Figure 3) were integrated into the search engine of a newly developed bioinformatics package named ‘TargetSiteAnalyzer’. TargetSiteAnalyzer was used to screen the entire human genome for functional genomic *FRT*-like sequences.

### TargetSiteAnalyzer

**Figure 4:**
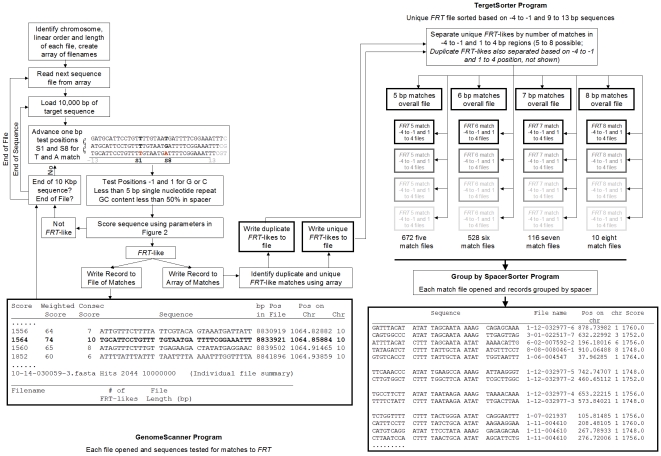
Overview of TargetSiteAnalyzer bioinformatics package used to find *FRT*-like sequences within 2.8 Gbp of human genomic DNA (NCBI build 36.3). Heavy-bordered boxes indicate the creation of a file. File format boxes may have spaces added for readability in this figure.

TargetSiteAnalyzer is composed of three JAVA programs that are sequentially run: *GenomeScanner*, *TargetSorter* and *SpacerSorter* (Text S1, S2, S3, S4). Together, these programs perform the task of identifying and then sorting *FRT*-like sequences within a genome of interest. An overview of these programs and the processing steps is shown in Figure 4.

*GenomeScanner* sequentially screens each DNA contig file within a genome build for *FRT*-like sequences using the rules that describe sites that can serve as functional recombination targets. A contig file is successively read as overlapping 34-nucleotide segments in 1-nucleotide increments. Each 34-nucleotide sequence is separated into three regions (Figure 3): two potential inverted recombinase-binding elements (positions −13 through −1 and positions 1 through 13) and a spacer (positions s1 through s8). As does TargetFinder [14], *GenomeScanner* first checks if a putative spacer has a ‘T’ at position s1 and an ‘A’ at position s8 and whether GC content of the spacer equals or is below 50%. These criteria for a functional spacer are based on the observations that *FRT*-like sequences with such spacers support efficient recombination [17]. If criteria for a functional spacer are met, *GenomeScanner* tests positions −4 to −1 and 1 to 4 and also −7 and 7 of the putative binding elements of an *FRT*-like sequence for the number of matches and mismatches to the corresponding positions of *FRT*. In addition, the entire 34-nucleotide sequence of an *FRT*-like site is tested for any single nucleotide repeat longer than four nucleotides. The putative binding elements of an *FRT*-like sequence are also checked for the number of consecutive matches (Figure 3). Each position in the binding elements of an *FRT*-like sequence (positions −13 to −1 and 1 to 13) that is matched to the corresponding position in *FRT*, is given a weighted value and a total score for an *FRT*-like sequence is generated that includes the number of matches within the ‘proximal-8’ sequence and the weighted value.

During program execution, *GenomeScanner* writes each match to a linear-order text file and to an internal array. After the last sequence file is processed, *GenomeScanner* uses the array to determine which *FRT*-like sequences are unique, then generates two additional output files: one containing only unique *FRT*-like sequences and a second containing *FRT*-like sequences with at least one exact duplicate. *GenomeScanner* reports the position of each identified *FRT*-like sequence both within the sequence contig files and within a chromosomal fragment map based on linear order of files for each chromosome and the cumulative base pairs for each chromosome (Figure S1).

*TargetSorter* works with the *GenomeScanner* generated files that contain both the unique and duplicated *FRT*-like sequences. The program groups the records based on the sequence of the most functionally important region of the *FRT* putative recombinase binding elements (−4 to −1 and 1 to 4). In this region, both complimentary strands are assigned a numeric value. The lowest value is used to assign the record to a file.

The *SpacerSorter* program sorts *FRT*-like sequences within each output file generated by *TargetSorter* based on spacer sequence. In similar fashion to the *TargetSorter* program, both directions of the spacer sequences are used to determine if a match exists. This final sorting step is important since it allows identification of those *FRT*-like sequences that can, in principle, recombine with each other by a single Flp variant specific for a particular sequence pattern in the ‘proximal-8’ region.

### Unique and repeated *FRT*-like sequences in human genome

**Figure 5:**
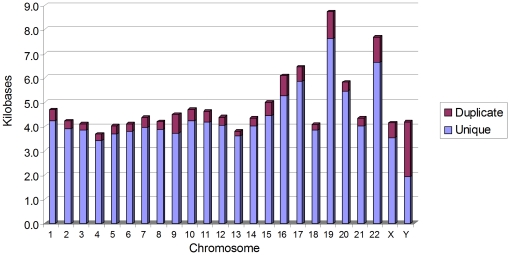
Average distance between *FRT*-like sequences in human chromosomes. The highest density of *FRT*-like sequences is in chromosome 4; the lowest density is in chromosome is 19. The ratio of unique to duplicated *FRT*-like sequences is indicated by the relative size of the bars.

*GenomeScanner* identified 642,151 potentially functional *FRT*-like sequences in the human genome (Text S5). Out of those, 581,157 *FRT*-like sequences are unique and 60,994 have at least one exact duplicate. In the majority of human chromosomes, unique *FRT*-like sequences can be found, on average, every 4 to 5 kb (Figure 5). Notable exceptions are chromosomes 19 and 22, which average *FRT*-like sequences every 9 to 10 kb and 8 kb, respectively. This average distribution of the *FRT*-like sequences correlates very well with GC content in human chromosomes [19], where chromosomes 4 and 13 have the lowest GC content (∼38%) and chromosomes 19 and 22 – the highest (∼48%). Since overall GC content of *FRT* is about 33% and GC content of a functional spacer in an *FRT*-like sequence should be equal or below 50%, the dependence of average distribution of *FRT*-like sequences on GC content in a genome of interest can be satisfactorily explained. Of note, thermophilic bacteria, with their high GC content, usually have only about one *FRT*-like sequence per 1 Mb of their genomes (Y.V., unpublished).

We did not investigate if there is a preference for *FRT*-like sequences to be in exons or introns in a gene per arbitrary unit of DNA length. We noted, however, an obvious preference for *FRT*-like sequences to be in introns (most likely because they are, on average, significantly longer than exons). It is possible, however, that there is a preference for *FRT*-like sequences for introns in general since, on average, GC content of introns is lower than that of exons [20]. Also, for the same reason of lower GC content, there might be a preference for *FRT*-like sequences to be in longer, rather than shorter introns, and in terminal and intermediate exons than in initial exons [20].

12,452 of the *FRT*-like sequences identified in the human genome have at least one exact duplicate. The copy number of duplicated sequences ranged from 2 to 6,387, bringing the total number of duplicated *FRT*-like sequences to 60,994. On average, about 10% of all *FRT*-like sequences in human chromosomes are duplicated, however, as can be seen in Figure 5, chromosome Y is exceptional. About 50% of *FRT*-like sequences in this chromosome are duplicated. This correlates well with the unusually high frequency (∼50%) of repeated DNA elements, particularly LINE1, in chromosome Y [21].

**Figure 6:**
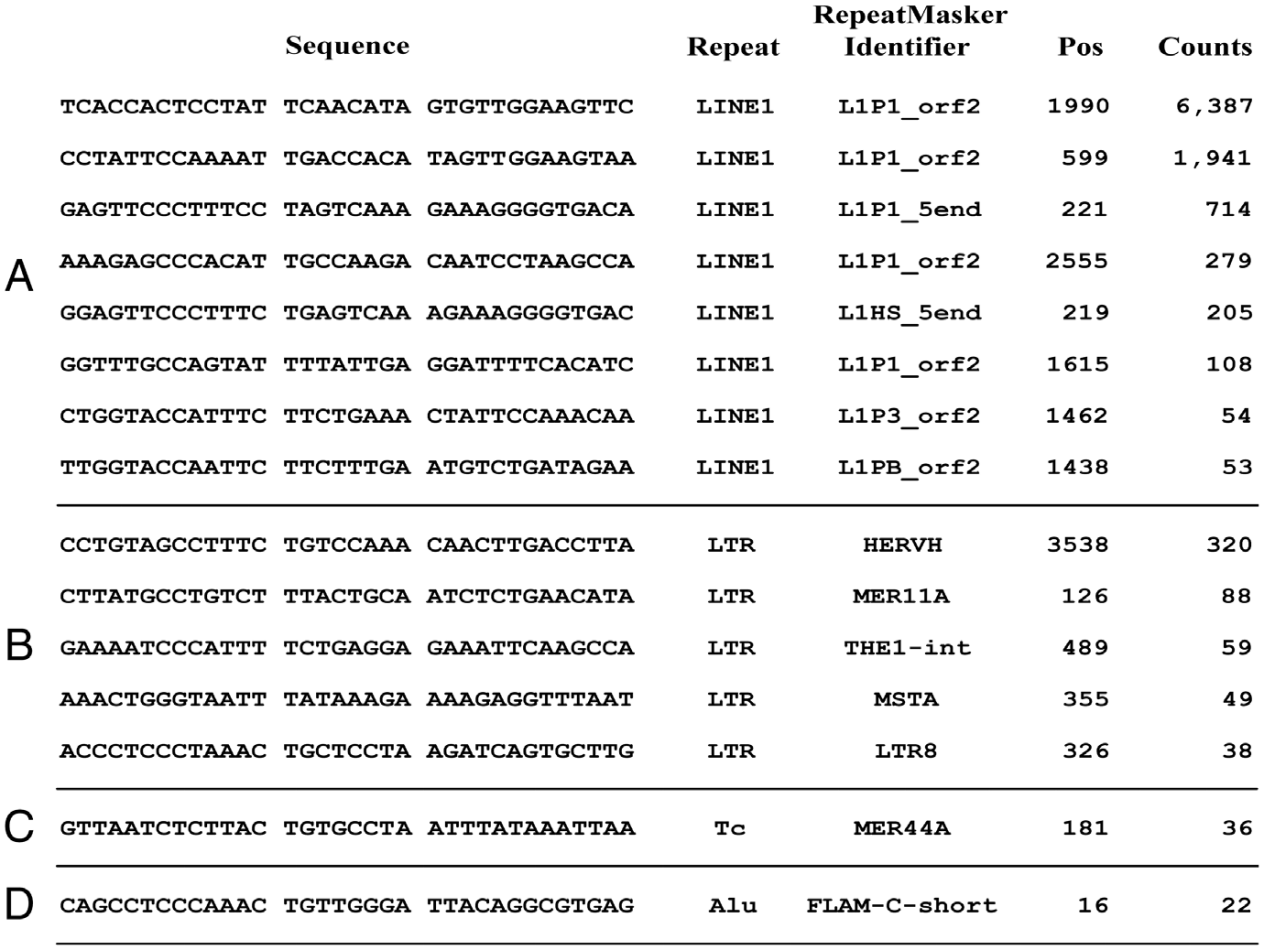
*FRT*-like sequences in repeated DNA sequences in human genome Examples of repeated *FRT*-like sequences in (**A**) LINE-1, (**B**) LTRs of endogenous retroviruses, (**C**) Tc element, (**D**) Alu repeats.

**Figure 7:**
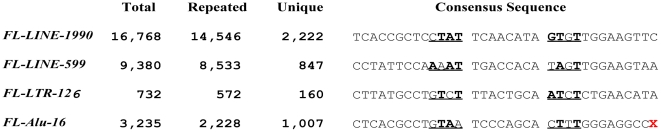
Distribution of some *FRT*-like sequences originated in repeated DNA elements between repeated and unique pools. *FL-LINE-1990* and *Fl-LINE-599* represent groups of the *FRT*-like sequences that originate at positions 1990 and 599 of ORF2 of LINE1, respectively (see Figure 6). *FL-LTR-126* represents a group of the *FRT*-like sequences that originate at position 126 of LTR of transposon MER11a (Figure 6). *FL-Alu-16* represents a group of the *FRT*-like sequences that originate at position 16 of the left half of Alu sequence (Figure 6). Consensus sequence for *FL-Alu-16* has 33 nucleotides; red “X” indicates any nucleotide.

In the vast majority of cases, duplicated *FRT*-like sequences are part of repeated DNA (as judged by RepeatMasker [www.repeatmasker.org]): mainly LINE1, but also LTRs of endogenous retroviruses, Alu sequences and some other repeats (Figure 6). The most frequently duplicated *FRT*-like sequence (6,387 copies) is found in LINE1 at position 1990 of ORF2 that codes for reverse transcriptase (Figure 6A). This sequence is present in all chromosomes averaging from one per 0.24 Mbp in chromosomes X and Y (Figure S2) to one per 1.9 Mbp in chromosome 18. There are multiple variations of this particular sequence in different copies of LINE1 repeats forming different groups of repeated *FRT*-like sequences originated at position 1990 of ORF2 (Figure 7). These groups of *FRT*-like sequences have lower copy numbers than the “main” one.

Other repeated *FRT*-like sequences also form groups (Figures 6 and 7). Members of these groups originate from the same sequence in a repeated DNA element but differ in one or more nucleotides from each other. Groups of repeated *FRT*-like sequences are seen in all repeated DNA elements analyzed: LINE1, LTRs, Alu sequences and others. Analysis shows that four *FRT*-like sequences that form the most populous groups in their categories (two from LINE1, and one each from LTRs and Alu; Figure 7) account for nearly half of all duplicated *FRT*-like sequences.

The number of the *FRT*-like sequences located in repetitive DNA elements in the human genome is underestimated since we noted that some *FRT*-like sequences, although unique, originate from the duplicated DNA elements and belong to the respective groups of repetitive *FRT*-like sequences (Figure 7).

### Classes and subclasses of unique *FRT*-like sequences with common sequence patterns

Since we found that the proximal–8 region is the most critical for determining the functional performance of a genomic *FRT*-like sequence, we grouped all unique *FRT*-like sequences (581,157 sites) based on the sequence of the first four proximal base pairs of their putative recombinase binding elements (positions −1 to −4 and 1 to 4; Figure 1A). Since, according to our search criteria, functional *FRT*-like sequences in the proximal-8 region can have 8, 7, 6 or 5 matches to the corresponding nucleotides of *FRT*, we first grouped all unique *FRT*-like sequences into four major classes based on the number of matches in this region. *FRT*-like sequences in each major class were then sub-classified based on the actual sequence of the proximal-8 region.

Although *FRT*-like sequences of the class ‘8’ have, by definition, only matching nucleotides in the proximal-8 region, this does not mean that there is only one possible sequence in this region for the class. Since the consensus sequence of *FRT* for the purpose of identifying functional *FRT*-like sequences, *FRT*-mod (Figure 1A), allows A or G at position 1 (and, therefore, C or T at position −1) and A or T at positions −2/2, there are 10 possible sequences in the proximal-8 region that perfectly match those in *FRT*-mod. All *FRT*-like sequences of the class ‘8’ were in fact sorted by the program into 10 subclasses. It should be noted that calculation of the number of subclasses in major classes takes into account both direct and inverse complement of a sequence in the proximal-8 region to eliminate those subclasses that seem to represent two different sequences but in fact are direct and inverse complement versions of the same sequence.

When both number of matched and the nature of mismatched base pairs within the proximal-8 region are taken into account, the number of subclasses for the classes 7, 6 and 5 were found to be 116, 528 and 672, respectively. Consequently, all unique *FRT*-like sequences of the classes 7, 6 and 5 were sorted into their respective subclasses (Text S6).

A final sorting was performed within each subclass: *FRT*-like sequences were sorted based on their spacer sequences (in both direct and inverse complement orientations) to identify those *FRT*-like sequences that have identical spacers and therefore can potentially recombine with each other (Text S7). A snapshot of a subclass file with *FRT*-like sequences sorted based on the sequence of their spacers is shown in Figure 4.

To help identify *FRT*-like sequences with similar sequences patterns, we also grouped all 12,452 of the *FRT*-like sequences that have at least one exact duplicate in the human genome. The grouping was performed the same way as for the unique *FRT*-like sequences: into major classes and subclasses (Text S8). Finally, duplicated *FRT*-like sequences were sorted within each subclass based on their spacer sequences (Text S9).

### *FRT*-like sequences similar to FRT-like sequences of interest

**Figure 8:**
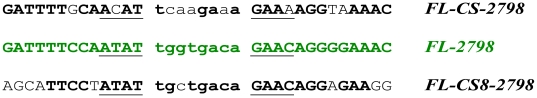
Two *FRT*-like sequences that are highly homologous to *FL-2798*. Identical nucleotides between the *FRT*-like sequences are shown in bold. Spacer sequences are shown in small letters. 8-proximal regions in the sequences are underlined. *FL-CS8-2798* has the same 8-proximal region as *FL-2798* but fewer overall identical nucleotides than has *FL-CS-2798*.

Identification of all potential *FRT*-like sequences in the human genome and their classification into classes and subclasses makes it easier to locate *FRT*-like sequences that have the highest level of homology to an *FRT*-like site of interest and that can serve as counter-selection targets during evolution of a Flp variant specific for this *FRT*-like site. To locate homologous *FRT*-like sequences, we developed an additional program called *CompareSequences* that scans all identified *FRT*-like sequences (unique and repeated) against an *FRT*-like site of interest and reports sequences that have their level of homology above a set value (Text S10). The output of the program also includes *FRT*-sequences from a subclass, to which the *FRT*-like site of interest belongs; the *FRT*-sequences from a respective subclass may not have the highest level of homology to the *FRT*-like site of interest but they have the perfect match in the functionally important proximal-8 region and, therefore, should be considered as the first choice for selecting a counter-selection target. As an example, we searched for counter-selection targets for the *FRT*-like site *FL-2798* (Figure 1B). The *FRT*-like sequences with the highest level of homology to *FL-2798*, identified by the *CompareSequences* program are shown in Figure 8. *FL-CS-2798* is from the general pool of the genomic *FRT*-like sites and *FL-CS8-2798* is from the proximal-8 subclass, to which *FL-2798* belongs. When only putative recombinase binding sites are considered, *FL-CS-2798* and *FL-CS8-2798* differ from *FL-2798* by five and eight nucleotides, respectively. The identified *FRT*-like sites can be used as counter-selection sequences during evolution of Flp variants able to recombine *FL-2798* with minimum off-target effects.

## Discussion

The main goal of the present work was to gain insight into sequence properties and distribution of *FRT*-like sequences in the human genome. To accomplish this goal we solved three tasks: (1) we developed a computer program able to quickly scan a mammalian genome for target-like sequences for tyrosine recombinases; (2) we analyzed genome-wide distribution of *FRT*-like sequences in the human genome, and (3) we sorted the identified *FRT*-like sequences into groups that have common sequence patterns.

### TargetSiteAnalyzer

The bioinformatics package dubbed ‘TargetSiteAnalyzer’ is able to quickly, within several hours using a regular office computer, search the entire human genome for *FRT*-like sequences and sort them according to their sequence properties. We deemed it useful to separate the in-silico analysis into three sub-programs. This separation allows the user to make changes to an algorithm without the need to re-run the entire analysis. The *GenomeScanner* program allows the processing of essentially infinite number and sized files by searching for *FRT*-like sequence within a small string of 10,000 bp, well within the memory parameters of most computers. *GenomeScanner* creates a large, single file with *FRT*-like sequences that have three different scores: overall, weighted and consecutive match scores; this allows the re-sorting of match data as experimental results indicate which parameters are most predictive of success. The *TargetSorter* program assigns a numeric value to the nucleotides in the proximal-8 region (Figure 1A) in order to identify and group similar *FRT*-like sequences irrespective of their orientation in a DNA contig. The *SpacerSorter* program allows the easy identification of those *FRT*-like sequence in a given subclass that have identical spacers. All Java codes are provided so that TargetSiteAnalyzer can be modified to search for and analyze other target sequences for site-specific recombinases.

To identify *FRT*-like sequences, TargetSiteAnalyzer utilizes a simple sequence scanning approach. In principle, other, more sophisticated genome analysis approaches could be used to accomplish the goal. Since *FRT*-like sites share conserved sequence patterns, these patterns could be identified using probabilistic sequence analysis approaches, such as a hidden Markov model, HMM [22], [23]. HMM could also be useful in grouping the identified *FRT*-like sequences.

### Functional *FRT*-like sequences in human genome

An important contribution of our work is the analysis of *FRT*-like sequences in the human genome. The analysis provides not only an overview of the distribution of the sequences but also their classification based on common sequence patterns. The analysis is a valuable resource for designing genome engineering experiments using tailor-made variants of Flp recombinases, since it assists in choosing *FRT*-like sequences in the vicinity of a genomic region of interest by taking into account which class and subclass it belongs to. By doing so, the closest *FRT*-like sequence that can be used as a counter-selection target during Flp variant evolution can be identified [11]. Such an approach to designing experiments would minimize off-target effects of evolved Flp variants.

In theory, for each unique *FRT*-like sequence, it should be possible to evolve a unique Flp variant that would preferentially recombine this sequence. The probability of finding a particular *FRT*-like sequence is ∼10^−21^ (or ∼10^−16^ if only recombinase-binding elements are considered). The size of the human genome is ∼3×10^9^ so it should be virtually impossible to find a duplicate for a given *FRT*-like sequence in the human genome unless this sequence is part of repeated DNA. Since roughly half of the human genome is repetitive DNA (which can bear potential *FRT*-like sequences), we, as a precaution, had to check if identified *FRT*-like sequences have duplicates. It appeared that our precaution was justified, as we found that around 10% of all *FRT*-like sequences in the human genome are duplicated and originated mainly from LINE1, LTRs of retrotransposons and Alu sequences. We also found that there are unique *FRT*-like sequences that originate from the same locations in the repeated DNA elements as the respective duplicated *FRT*-like sequences and, therefore, should belong to the ‘duplicated’ group even though they do not have additional exact copies.

As in unique genomic regions, we did not find any apparent ‘role’ or ‘function’ for the *FRT*-like sequences in the repetitive DNA in the human genome. Since we observed very good correlation between average distribution of the *FRT*-like sequences and GC content in human chromosomes (Figure 5; [19]), and found that *FRT*-like sequences are essentially absent in genomes of thermophilic bacteria (Y.V., unpublished), we can satisfactory explain the presence of *FRT*-like sequences in genomic localities with relatively low GC content (less than 50%).

Although it was unexpected to find that *FRT*-like sequences can originate from more than one location in LINE1 (Figure 6A), on average, *FRT*-like sequences are more rare in repeated genomic DNA than in unique DNA: repetitive DNA constitutes about 50% of the human genome but only about 10% of all *FRT*-like sequences are part of repeats. This observation is not surprising taking into account that the majority of repeated DNA elements are relatively short. Indeed, only infrequent full-length or near full-length units of LINEs or LTR transposons are long enough to have a high probability of occurrence of *FRT*-like sequences (Figure 5), while the majority of copies of LINEs are shorter than 1 kb and SINEs are only 100–400 bp long [24]. Moreover, since different members of a family of repeated DNA elements are usually not identical due to random mutations, one repeated element might have an *FRT*-like sequence but another might not.

Multiple identical copies of duplicated *FRT*-like sequences can, in theory, present a challenge for targeting unique *FRT*-like sequences of interest if the duplicated sequences have a high degree of homology to a chosen unique *FRT*-like site. In this scenario, a Flp variant evolved to recombine the unique *FRT*-like site could, to a certain degree, recombine the repeated *FRT*-like sequences. To prevent this and therefore minimize the off-target effects of the recombination system, the repeated *FRT*-like sequence should be used as one of the counter-selection targets during evolution of a Flp variant.

To improve our understanding of the sequence properties of the unique *FRT*-like sequences and to help identify the ones with similar sequence patterns, we grouped both the duplicated and unique *FRT*-like sites into classes based on the number of matches (8, 7, 6, or 5) to the corresponding positions of *FRT* in the proximal-8 region (Figure 1A). This region contains the most important, but not all determining factors for successful recombination of a genomic *FRT*-like sequence. The least populous and the closest to *FRT* is the 8-match class, which has 8 matches in the proximal-8 region. The most populous and the least close to *FRT* is the class 5, which have 5 matches in the proximal-8 region.

Classification of the *FRT*-like sequences helps identify *FRT*-like sites which have the highest level of homology to an *FRT*-like site of interest and which can serve as counter-selection targets during evolution of Flp variants specific for this *FRT*-like site. Ideally, the counter-selection *FRT*-like sequences should come from a subclass, to which an *FRT*-like site of interest belongs, since all sequences in this subclass have identical functionally important proximal-8 region. However, due to a relatively low number of the *FRT*-like sequences in this particular subclass, it is unlikely that it contains an *FRT*-like sequence that has the highest level of homology to an *FRT*-like site of interest. Therefore, in an effort to have a broader picture, the computer program *CompareSequences*, which we developed to identify homologous *FRT*-like sequences, scans all genomic *FRT*-like sequences. From the output of the program, two *FRT*-sequences can be chosen as counter-selection targets: one from the subclass to which the *FRT*-like sequence of interest belongs, and one from the rest of the *FRT*-like sequences. The counter-selection *FRT*-like sequences will help evolve the most specific Flp variants able to recombine the *FRT*-like site of interest.

### Conclusion

Our work is the first genome-wide analysis of the distribution and sequence properties of target-like sequences for a site-specific recombinase in the human genome. We found that *FRT*-like sequences are located in the human genome roughly every 5 kb. The identified *FRT*-like sequences were divided into two groups, which contain either unique or duplicated sequences. The duplicated *FRT*-like sequences originate mainly from such repeated DNA elements as LINE1, LTRs and Alu repeats. All *FRT*-like sequences were subdivided into classes and subclasses based on their sequence features. The database of genomic *FRT*-like sequences created can be used to identify *FRT*-like sequences that can serve as counter-selection sites for evolving a Flp variant specific for an *FRT*-like sequence of interest. Our data will be useful for the emerging field of genome engineering.

## Materials and Methods

### Human genome DNA

Contig files containing entire assembled human genome (NCBI build 36.3) were downloaded from NCBI web site: http://www.ncbi.nlm.nih.gov/genomes/genlist.cgi?taxid=2759&type=8&name=EukaryotaeCompleteChromosomes. Each of 473 files totaling 2.837 Gbp of DNA was identified by chromosome and order, allowing the alignment of contiguous DNA fragments from each chromosome. A map of these ordered DNA fragments is presented in Figure S1.

### TargetSiteAnalyzer Development and Execution

The process is executed as three individual programs: *GenomeScanner*, *TargetSorter*, and *SpacerSorter*. An overview of these programs and the processing steps is shown in Figure 4. The programs were created and executed within the freely available NetBeans IDE (version 6.8; http://netbeans.org). The JAVA code is available for download (Text S1, S2, S3).

*GenomeScanner* is designed to read all DNA sequence files (in FASTA format) in a directory, create an array of sequence contig filenames, then process each sequence file base-by-base to identify sequences that match a particular profile. The first step in the process is to identify the sequence file and set the chromosome and location of the sequence within the file within context of the chromosome. Each sequence file is read line by line and added to the end of a string, until the string reaches 10,000 bp in length. Each of these strings is tested for a match to the *FRT* sequence by advancing 1 base, acquiring the 34 base sequence, then testing it as indicated below and as shown in Figures 3 and 4. Once the string length from the last position tested equals 39 bp, the next lines from the sequence file are added until the string again reaches 10,000 bp and the process is repeated.

Once a potential *FRT*-like sequence is identified, it is written along with information such as file name, scores, position within the sequence file and overall position on the chromosome to an internal array and a linear order text file (which allows identification of nearby *FRT*-like sequences regardless of sequence structure). At the end of the last sequence file, the internal array of the *FRT*-like sequences is used to identify exact forward and reverse matches within all identified *FRT*-like sequences and write these repeated sequences to a separate file. All other *FRT*-like sequences that do not have an exact match are written to a file of unique sequences. *GenomeScanner* has 2,795 lines of code, of which 1,892 use the file name of each of the 473 human genome contig files (NCBI build 36.3) to identify the chromosome and an offset number used for positioning each *FRT*-like sequence within the linear chromosome sequence. Using a regular office computer, this program executes in its entirety in approximately 470 minutes, or 1 minute per input file.

Using either the file with unique or repeated *FRT*-like sequences created by the *GenomeScanner* program, *TargetSorter* creates general files that contains all *FRT*-like sequences with the same number of matches within the proximal-8 region. Within each file, the nucleotides at the proximal-8 region are assigned a number with A = 1, T = 2, G = 3 and C = 4. For example, if a sequence in the proximal-8 region is ATAC-spacer-AATA, its number is 12,141,121; the number of the inverse complement of this proximal-8 region sequence (TATT-spacer-GTAT) is 21,223,212. The numbers in both the forward and reverse complement orientation of the proximal-8 region are compared, with the lowest value used for the filename designation. Each resultant file contains only *FRT*-like sequences that belong to a unique subclass. *TargetSorter* has 332 lines of code. The program execution speed varies based on input file size, taking approximately one hour to sort the 581,157 unique *FRT*-like sequences.

Each subclass-specific file created by *TargetSorter* is processed by the *SpacerSorter* program, which compares all spacer sequences within the file and then creates a new file, which lists *FRT*-like sequences with identical spacers first, each group of sequences delineated by a blank line, followed by a list of the *FRT*-like sequences with unique spacer sequences. Subclass-specific files containing repeated *FRT*-like sequences by definition do not have *FRT*-like sequences with unique spacers. Figure 4 shows an example output from *SpacerSorter*. This program has 154 lines of code and typically executes in 1 minute or less when processing the 1,325 files containing subclass-specific unique *FRT*-like sequences.

The *CompareSequences* program scans a group of files containing all unsorted *FRT*-like sequences (both unique and repeated) for the *FRT*-like sequences homologous to an *FRT*-like site of interest. The level of homology is determined by the number of nucleotides that are identical in a given *FRT*-like sequence and in the *FRT*-like site of interest. *CompareSequences* reports the *FRT*-like sequences that have the number of identical nucleotides above the set threshold. The program also reports all *FRT*-like sequences from the subclass file, to which the *FRT*-like site of interest belongs, indicating the number of identical nucleotides between an *FRT*-like sequence and the *FRT*-like site of interest.

### Other methods

The Flp-DNA structure (PDB code 1FLO) was analyzed using Swiss-PdbViewer [25]. General genetic engineering experiments were performed as described in Sambrook and Russell [26]. Molecular evolution of the Flp variant genes was performed as described in Bolusani et al. [14]. The evolved Flp variants were tested using the lacZα deletion reporters [11], in which the lacZα cassette was flanked by an *FRT*-like sequence of interest and *FRT* in which its native spacer was replaced with a spacer from the *FRT*-like sequence.

To construct the pEGFP-del reporter, the EGFP gene was PCR-amplified from the pIRES2-EGFP vector (Clontech) with primers that contained either *FL-61204* or *FL-63904* or *FL-71362* sites (forward primer) and a modified *FRT* site (reverse primer). The amplified EGFP gene was cloned into pcDNA5/FRT (Invitrogen) under the control of the CMV promoter between NheI and HindIII to obtain pcDNA5-EGFP. Then, the DsRed gene from pIRES2-DsRed-Express (Clontech) was PCR-amplified and cloned into pcDNA5-EGFP between BamHI and XhoI to obtain pEGFP-del.

The mammalian expression vectors for Flp variants were constructed by cloning the genes for the variants into pOG44 (Invitrogen) in place of the *FLP*-F70L variant gene.

Chinese Hamster ovary cells (CHO-K1, ATCC CCL-61) were propagated in F12-K media. A Flp recombinase variant expressing vector and a respective pEGFP-del reporter were co-transfected into CHO cells with a ratio of 8∶1 (w/w). Transfections were performed using Polyfect (Qiagen) according to the manufacturer's recommendations.

## Supporting Information

Figure S1 **Map of contiguous DNA fragments in NCBI build 36.3 of human genome.** File names are listed to the right of each chromosome. The map was generated using the MapChart program. The map can be viewed and magnified using, for example, the Windows Picture and Fax Viewer program.
(EMF)

Figure S2 ***FRT*** **-like sequences on human chromosomes X and Y.** Black horizontal bands within chromosomal bars indicate unique *FRT*-like sequences; red horizontal bands indicate repeated *FRT*-like sequences. Each number bar on the left panel corresponds to 10,000 base pairs. The map was generated using the MapChart program. The map can be viewed and magnified using, for example, the Windows Picture and Fax Viewer program.
(EMF)

Text S1 Java code for *GenomeScanner* program.
(TXT)

Text S2 Java code for *TargetSorter* program.
(TXT)

Text S3 Java code for *SpacerSorter* program.
(TXT)

Text S4 TargetSiteAnalyzer Installation and Run Parameters.
(DOC)

Text S5 All *FRT*-like sequences in human genome identified by *GenomeScanner* program.
(TXT)

Text S6 Example of a file from the pool of files of unique genomic *FRT*-like sequences sorted into classes and subclasses. Zip archive of the pool can be downloaded from http://www2.latech.edu/~voziyan/index_files/TextS6.zip.
(TXT)

Text S7 Example of a file from the pool of files of unique genomic *FRT*-like sequences that have their spacers grouped within each subclass file. Zip archive of the pool can be downloaded from http://www2.latech.edu/~voziyan/index_files/TextS7.zip.
(TXT)

Text S8 Example of a file from the pool of files of duplicated genomic *FRT*-like sequences sorted into classes and subclasses. Zip archive of the pool can be downloaded from http://www2.latech.edu/~voziyan/index_files/TextS8.zip.
(TXT)

Text S9 Example of a file from the pool of files of duplicated genomic *FRT*-like sequences that have their spacers grouped within each subclass file. Zip archive of the pool can be downloaded from http://www2.latech.edu/~voziyan/index_files/TextS9.zip.
(TXT)

Text S10 Java code for *CompareSequences* program.
(TXT)
