# Supplementary material for: A Genome-Wide Analysis of FRT-Like Sequences in the Human Genome
Source: PLoS One. 2011 Mar 23;6(3):e18077. doi: 10.1371/journal.pone.0018077 (PMC3063242; doi:10.1371/journal.pone.0018077)
Supplement: Text S4 — TargetSiteAnalyzer Installation and Run Parameters. (DOC) [file pone.0018077.s006.doc]

**Genome Scanner Program**

Program Setup Instructions and Files Required

The first time you run the program you will need to create a new project. Select "FILE", then "New Project", then "JAVA". The program will ask you for a project name, enter "**GenomeScanner**" then select finish. Copy all of the program code into this window, eliminating all other code. Press the green arrow to run.

473 FASTA formatted files from Build 36.3 of the Human Genome must be in the following directory

**"c:\\genome processing files\\human DNA"**

Your output will be placed in a file in the following directory

**"c:\\genome processing files"**

You will be asked for the name of your project

You will also be asked for the time - this helps to create a unique filename - you can also enter a date in this field which will be placed in the output filename

**Target Sorter Program**

Program Setup Instructions and Files Required

The first time you run the program you will need to create a new project. Select "FILE", then "New Project", then "JAVA". The program will ask you for a project name, enter "**TargetSorter**" then select finish. Copy all of the program code into this window, eliminating all other code. Press the green arrow to run.

The complete file name and path for either the duplicated or unique file created by the GenomeScanner program must be identified in a pop-up screen.

Your output will be placed in a file in the following directory

**"c:\\genome processing files\\FRT files\\"**

**Spacer Sorter Program**

Program Setup Instructions and Files Required

The first time you run the program you will need to create a new project. Select "FILE", then "New Project", then "JAVA". The program will ask you for a project name, enter "**TargetSorter**" then select finish. Copy all of the program code into this window, eliminating all other code. Press the green arrow to run.

Copy the TargetSorter output files from the **"c:\\genome processing files\\FRT files\\"** directory to the **"c:\\genome processing files\\FRT files\\Working\\"** directory.

These files are processed in the same directory and “sorted” is added to the file name.

**Compare Sequences Program**

Program Setup Instructions and Files Required

The first time you run the program you will need to create a new project. Select "FILE", then "New Project", then "JAVA". The program will ask you for a project name, enter "**CompareSequences**" then select finish. Copy all of the program code into this window, eliminating all other code. Press the green arrow to run.

All unsorted FRT files must be in the following directory

**"c:\\genome processing files\\FRT files\\Working\\"**

Your output will be placed in a file in the following directory

**"c:\\genome processing files\\FRT files\\"**

You will be asked for the number of matches to use as a cutoff

You will also be asked for the time - this helps to create a unique filename - you can also enter a date in this field which will be placed in the output filename

The first time you run the program you will need to create a new project. Select "FILE", then "New Project", then "JAVA". The program will ask you for a project name, enter "**searchspecificsequence**" then select finish. Copy all of the program code into this window, eliminating all other code. Press the green arrow to run.
